# Stable angina in young women

**DOI:** 10.1093/eurheartj/ehaf728

**Published:** 2025-10-07

**Authors:** Carolyn M Webb, Peter Collins

**Affiliations:** National Heart & Lung Institute, Imperial College London, and Royal Brompton Hospital, Sydney Street, London SW3 6NP, UK; National Heart & Lung Institute, Imperial College London, and Royal Brompton Hospital, Sydney Street, London SW3 6NP, UK

**Keywords:** Young women, Stable angina, Ischaemic heart disease, INOCA, ANOCA, Risk factors

## Abstract

Ischaemic heart disease (IHD) is the primary cause of cardiovascular death worldwide. Worryingly, the prevalence of IHD is increasing in younger women, with a 3% increase between 1990 and 2019. Although global IHD death rates have decreased in younger women overall, IHD mortality in younger women is increasing in certain high-income countries. Angina is the primary presenting symptom of suspected IHD and coronary artery disease. The presence of angina in patients with stable coronary artery disease is associated with an increased risk of major cardiovascular events compared with those without angina. Angina in young women is linked to future premature IHD events. There are particular features to consider in younger women presenting with stable angina—anginal symptom characterization may align with older women, but angina may occur cyclically in younger women and a similar pattern may be evident on diagnostic testing. Pathophysiologic mechanisms for angina in some young women may involve effects of ovarian hormones on vascular beds of the cardiovascular system. Traditional IHD risk factors are common to women and men; however, there are certain sex differences in their relevance to IHD risk, as well as influences that female hormones and reproduction have over a life course that affect IHD risk. In this review, we will explore stable angina in young women, consider possible pathophysiological mechanisms involved in their symptom presentation, and present risk factors associated with IHD in women. In addition, potential treatment options will be discussed and attention will be drawn to gaps in the evidence, proposing areas where more research is necessary.

## Graphical Abstract

**Graphical Abstract:**
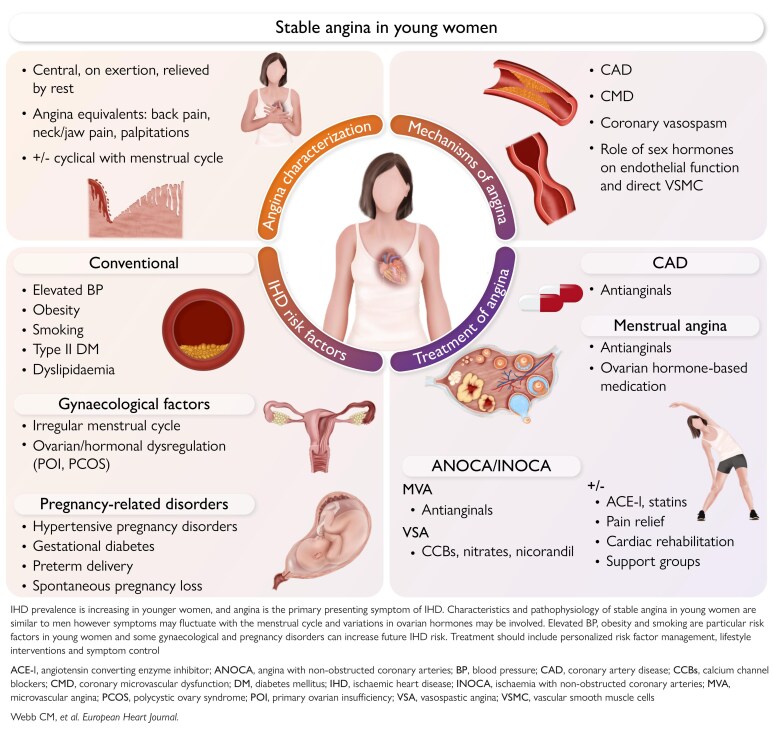

## Introduction

Cardiovascular disease (CVD) is a major cause of morbidity and mortality globally. In the majority of regions of the world, ischaemic heart disease (IHD) is the primary cause of cardiovascular death in women and in men^1^ and its prevalence is increasing in younger women. An analysis of the Global Burden of Disease dataset showed a 3% increase in the prevalence of IHD in younger women between 1990 and 2019 women, with the most significant increase in high-middle-income regions.^2^ Conversely, global death rates from IHD in younger women have decreased overall, although mortality from IHD in younger women is increasing in certain high-income countries such as the USA and Canada,^3^ though mostly related to smoking and obesity.

Angina is the primary presenting symptom of suspected IHD and atherosclerotic coronary artery disease (CAD) in women and in men.^4^ The prevalence of angina in both women and men, and the associated economic burden, is substantial.^5–7^ In 2017–20, the prevalence of angina in the USA was 10.8 million, 4.3% in males and 3.6% (5.2 million) in females.^8^ In England, angina accounted for 2.7% of cardiovascular conditions (an estimated 1.4 million people), 3.2% in men and 2.1% in women.^7^ A meta-analysis of angina prevalence in 31 countries found a notable similarity in angina prevalence in women across nations, with a marginally higher prevalence in women compared with men.^9^ Diagnosing and treating stable angina requires substantial healthcare utilization and costs. Whilst high-risk patients cost substantially more over the short term, low-risk patients result in higher lifetime costs as they have a greater life expectancy.^5^ There is a scarcity of evidence describing the prevalence and incidence of angina in premenopausal women.

Stable angina is defined as pain or discomfort usually in the chest, sometimes radiating to the neck and jaw, that occurs with activity, emotion or stress.^10^ ‘Angina equivalents’, symptoms that may occur instead of or in addition to angina, include isolated back, neck or jaw pain, breathlessness, fatigue, syncope, or palpitations—these characteristics tend to differ in prevalence by sex with women presenting more often with isolated back, neck or jaw pain, and palpitations and men with fatigue or/and weakness.^4^ Symptoms are usually, but not always, caused by a reversible mismatch of myocardial oxygen demand and supply associated with myocardial ischaemia or hypoxia caused by significant atherosclerotic epicardial CAD, coronary artery spasm, and/or microvascular dysfunction (MVD).^11^ On invasive testing for chest pain symptoms, approximately 50% of women will have obstructive coronary atherosclerosis compared with 60%–70% of men, and women more often have a microvascular pathophysiology to their symptoms.^11^ A recent comparison of computed tomography and invasive coronary angiography showed similar detection of CAD in women and men, but with both tests, women were less likely to have obstructive or/and severe anatomy.^12^ The presence of angina in patients with stable CAD is associated with an increased risk of major cardiovascular events such as heart failure, cardiovascular hospitalization, and coronary revascularization compared with those without angina.^13^ In this review, we will explore stable angina in young women and consider possible pathophysiological explanations for their symptom presentation and their risk factors associated with IHD. We will then discuss potential treatment options and draw attention to gaps in the evidence, proposing areas where more research is necessary.

Defining ‘young’ when discussing stable angina and IHD in women is complex and varies in the literature. Angina/IHD and menstrual or menopausal status are interconnected as we will discuss below—for instance, women generally have a delayed presentation of angina/IHD, compared with men, of approximately 10 years following their last menstrual cycle. A recent review of IHD in young women defined ‘young’ as <55 years^5^—this age range allows for the global variations in the average age of both the menopause and CVD risk depending on global region, ethnicity, and socio-economic status.^6^ Therefore, the definition for ‘young’ for the purposes of our discussion will be <55 years.

## Stable angina in young women

The incidence and prevalence of stable angina in young women are relatively unstudied; however, a recent examination of primary care data in the UK showed a flattening of the decline in the incidence and prevalence of angina (and IHD) in young women.^7^ In 2017, the annual incidence of angina in young women was 7.7 per 100 000 person years and the prevalence 51.5 per 100,000, with the average per cent change in incidence decreasing from −6.6% between 1998 and 2002 to −1.9% from 2007 to 2017.^7^ Overall, prevalence trends for angina and IHD paralleled incidence trends.^7^ Angina in young women is linked to future premature IHD events; a long-term follow-up of women aged 40–49 years, with angina but no evidence of IHD at baseline, revealed a doubling of the future risk of dying from IHD.^8^ Together, these data highlight the importance of acknowledging that young women presenting with angina should be taken seriously and be appropriately diagnosed and treated, and they also underscore the need for more contemporary data in this field.

The nature of presenting symptoms in women with stable angina include pain or discomfort usually in the chest that occurs with activity, emotion or stress.^10^ ‘Angina equivalents’, symptoms that may occur instead of or in addition to angina, typically present as isolated back, neck or jaw pain, and palpitations in women, compared with fatigue or/and weakness in men.^4^ The varied nature of presenting symptoms, together with a possible tendency to minimize symptoms or/and have concerns for family, may lead to delays in women seeking care for their anginal symptoms.^9^ In the acute setting, time from symptom onset to emergency department can be longer in women than in men with detrimental effects on 30-day mortality.^14^ Women with stable angina face the challenge of achieving an accurate diagnosis on initial presentation, considering differences in presentation and traditional and non-traditional IHD risk factors.^9^ There is no evidence currently to suggest that younger women present differently to older women; however, there are particular features to consider in younger, premenopausal women presenting with angina.

### Menstrual angina

The menstrual cycle, the recurring process that prepares a woman’s body for potential pregnancy, typically lasts 28 days and comprises a follicular phase (high oestradiol, low progesterone) and luteal phase (low oestradiol, high progesterone). It is known to affect medical conditions such as migraine, epilepsy, asthma rheumatoid arthritis, and irritable bowel syndrome,^15^ along with cardiovascular conditions such as coronary spasm, spontaneous coronary dissection, acute coronary syndrome, and cardiac arrhythmias in young women.^16–25^ Numerous case reports describe menstrual angina, often caused by coronary vasoconstriction, where cyclical variations in ovarian hormones related to the menstrual cycle were coincident with the onset of symptoms.^17,18,26,27^ In many cases, symptoms were refractory to conventional vasodilator treatment, leading to a hormonal cause being investigated. Tweet *et al*.^21^ observed chest pain that occurred during the day or two prior to the onset of menstruation in young women with a history of spontaneous coronary artery dissection, a condition that occurs significantly more frequently in women than men.^28^ These case reports and early research observations provide the foundations for further research into the possible role of the menstrual cycle and its associated hormonal fluctuations on symptoms in at least some young women with stable angina.

### Myocardial ischaemia and the menstrual cycle

**Figure 1:**
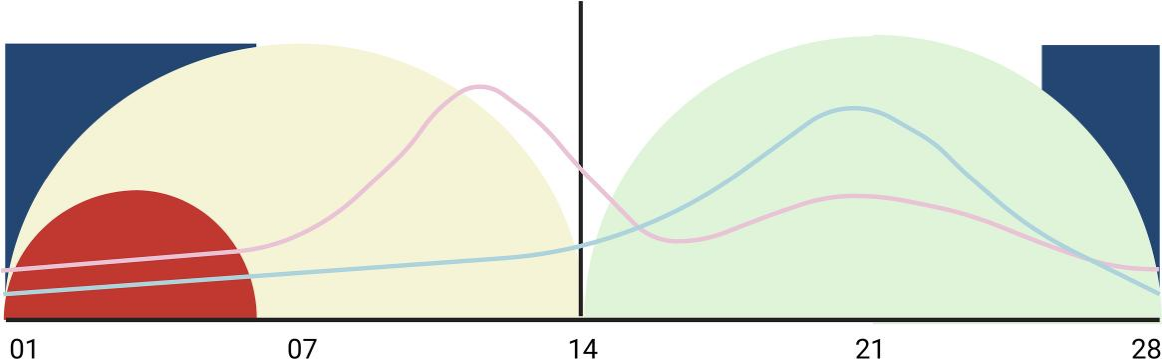
Hormonal variations associated with phase of the menstrual cycle and effects on angina, myocardial ischaemia, and flow-mediated dilatation. The menstrual cycle typically lasts 28 days and comprises follicular phase (yellow-shaded area; high oestradiol, low progesterone) and luteal phase (green-shaded area; low oestradiol, high progesterone). Numerous reports describe onset or increased frequency or severity of anginal symptoms, myocardial ischaemia, and decreased flow-mediated dilatation in the days immediately prior to or during the menstrual phase of the cycle (blue-shaded areas). Conversely, ischaemic episodes were least prevalent, and flow-mediated dilatation increased, in the follicular phase. Some of the evidence is preliminary however and contemporary studies in this area are needed. Oestrogen, pink line; progesterone, blue line. Numbers indicate day of the menstrual cycle; ovulation typically occurs on or around Day 14

Although contemporary practice favours computed tomography angiography as a first-line diagnostic test, exercise testing remains a useful investigation for reproduction of symptoms.^11^ Signs of myocardial ischaemia on exercise testing have been associated with menstrual cycle-related variations in ovarian hormones in women with stable angina, with and without obstructive epicardial CAD.^19,29,30^ In premenopausal women with vasospastic/variant angina, ischaemic episodes were most prevalent at the end of the luteal phase (low oestradiol, high progesterone) compared with the beginning of the menstrual phase and were coincident with reduced flow-mediated (endothelium-dependent) dilatation.^19^ Conversely, ischaemic episodes were least prevalent, and flow-mediated dilatation increased, in the follicular phase of the menstrual cycle when plasma progesterone concentrations were low and oestradiol was relatively high.^19^ Similar findings were reported in premenopausal women with ‘typical’ angina, a positive exercise test for myocardial ischaemia and unobstructed coronary arteries, where progesterone concentrations were found to be an independent factor influencing myocardial ischaemia, and time to angina on exercise was most prolonged in the late follicular phase (high oestradiol and low progesterone plasma concentrations) of the menstrual cycle.^29^ Likewise, in young women with symptomatic angina and established IHD, signs of myocardial ischaemia on exercise stress testing were more severe immediately prior to the onset of menstruation when oestradiol and progesterone were at their lowest concentrations.^30^ Although we acknowledge the preliminary nature of some of the evidence and the need for contemporary studies in this area, the available data indicate that timing the evaluation of anginal symptoms in young women may be important, keeping in mind that symptomatic and functional test results may vary depending on the phase of the menstrual cycle (*Figure 1*). Clinicians assessing young women with angina could at least note their current phase of the menstrual cycle.

### Angina with no obstructive coronary arteries

Approximately half of patients undergoing investigation of angina will have no significant epicardial CAD, but many will have coronary microvascular dysfunction (CMD), endothelial dysfunction, or epicardial coronary spasm on coronary functional testing.^31^ Ischaemia with no obstructive coronary arteries (INOCA) is diagnosed in those with proven myocardial ischaemia, with CMD and/or epicardial coronary artery spasm as underlying causes.^32^ Clinically, CMD results in microvascular angina (MVA) in patients with angina with no obstructive coronary arteries (ANOCA)/INOCA, but CMD and MVA can occur in patients with obstructive CAD also.^32^ Presentation can be diverse including conventional signs and symptoms and/or angina equivalents, and these symptoms may vary over time.^32^ The condition is heterogeneous, and evidence shows an increased risk of adverse cardiovascular outcome in those with CMD.^33,34^

ANOCA and INOCA more commonly occur in women than in men—50–70% of women undergoing coronary angiography have non-obstructive CAD—and women with non-obstructive CAD are younger than those with obstructive CAD.^32,33,35^ An investigation of the effect of age on ANOCA reported more severe angina in younger individuals (aged <45 years, 91% women) and a reduction of functional capacity with age.^36^ The higher incidence of symptoms in premenopausal women with ANOCA/INOCA may be due to an effect of hormonal fluctuations on the coronary vasculature.^37^

## Mechanisms

The menstrual cycle is regulated by the endocrine system via gonadotrophins and ovarian hormones, namely, oestrogens and progestins. Oestrogens are known to affect arterial vasoreactivity and blood flow in vascular beds beyond the reproductive system and may also affect pain perception. Unique risk factors for angina, IHD/CAD, or/and coronary MVD in young women revolve around not only their monthly reproductive cycles, as discussed above, but occur throughout their reproductive lives. In the following discussion, we will consider the evidence and the relevance of these factors to young women with angina.

### Ovarian hormones

#### Vasomotion and blood flow

Oestradiol-17β, the predominant and most potent oestrogen, is primarily synthesized in the ovaries and reversibly binds to sex hormone-binding protein in serum.^38^ It acts via oestrogen receptors α and β, classically via a nuclear receptor, but may also have rapid, non-transcriptional effects via oestrogen receptors, including the more recently identified G protein-coupled oestrogen receptor 1 (GPER), on the cellular plasma membrane.^39^ Oestrogen receptors are found throughout the cardiovascular system of both men and women, including in coronary arteries of premenopausal women, where oestrogen receptor expression is reduced in atherosclerotic compared with non-atherosclerotic arteries.^40^

The endothelium, a single layer of cells which line all blood vessels, mediates many vascular functions via substances such as nitric oxide (NO) and endothelin-1, among others. Oestrogen can stimulate NO production and potentiates arterial blood flow via endothelium-dependent mechanisms.^41,42^ Dysfunction of the endothelium precedes CVD development, with declining endothelial vasodilator function reportedly taking place across the menopause transition independent of chronological age and related to oestrogen deficiency.^43^ Theoretically, menstrual angina or menstrual vasospastic angina could be associated with coronary endothelial dysfunction related to the reduced oestrogen plasma concentrations associated with the menstrual phase of the menstrual cycle. This is yet to be proven; however, there is evidence for cyclical variations in flow-mediated (endothelium-dependent) vasodilation associated with phase of the menstrual cycle and related variations in oestradiol concentrations in the peripheral circulation of premenopausal women (*Figure 1*).^44,45^ A further study reveals variations in expired NO production with cyclical hormone changes in premenopausal women, with NO levels peaking at the middle of the menstrual cycle, suggesting an effect of ovarian hormones on the synthesis and/or release of NO.^46^ Oestrogen can inhibit vasoconstrictors such as endothelin-1 in the coronary circulation of postmenopausal women,^47^ a mechanism that may have relevance to women with menstrual angina where subtly raising serum oestrogen concentrations may reduce symptoms.^17,18,26,27^ Whilst there have been no studies investigating the effect of ovarian hormones on coronary endothelium-mediated vascular reactivity and blood flow in premenopausal women, evidence from postmenopausal women suggests a positive effect of oestradiol on vasomotor function in atherosclerotic epicardial coronary arteries and the coronary microcirculation via the endothelium.^48,49^ However, whether the cyclical, receptor-dependent nature of ovarian hormonal function in premenopausal women is likely to have similar effects on coronary vasoreactivity and blood flow to exogenous oestradiol in postmenopausal women is an area for further study. The data suggest a role for the vascular endothelium in the mediation of oestrogen’s effects in the peripheral and coronary circulations, but there are gaps in the evidence particularly relating to effects in premenopausal women.

As well as endothelium-mediated effects, oestrogen can act directly on the arterial vascular smooth muscle via an antagonistic effect on calcium channels.^50,51^ Recent findings describe a regulatory role of oestrogen on β-adrenergic receptors in cardiovascular tissues, with oestrogen receptors and β-adrenergic receptors acting as coregulators of cardiac calcium-handling proteins.^52^ There are no data that have progressed these findings into the clinical setting, but it is an intriguing area for potential future research.

#### Pain perception

Gonadal hormone receptors are found throughout the nervous system and are known to interact with nociception in the peripheral and central nervous systems.^53^ Clinically, the menstrual cycle can modulate the perception of migraine pain^54,55^; however, studies of experimental pain show inconsistent results.^53^ Medications such as imipramine, which blocks noradrenaline reuptake thereby enhancing the inhibitory action of pain-modulating neurons, can effectively treat symptoms of patients with ANOCA irrespective of the underlying pathology.^56^ There is no evidence to support the use of imipramine in young women with angina but it could be an interesting area for further investigation.

### Ischaemic heart disease risk factors particular to young women

Traditional risk factors for IHD—hypertension, hyperlipidaemia, diabetes, obesity, and cigarette smoking—are common to both men and women; however, there are features of each risk factor that are distinct for women (*Table 1*). Of these, hypertension is the leading global risk factor for CVD morbidity and mortality in women.^1^ A large sex-specific analysis of the trajectory of blood pressure over a life-course revealed that blood pressure elevation commences earlier in life—as young as aged 30–40 years—and rises more rapidly in women than in men.^57^ Women more often than men suffer the consequences of long-standing hypertension such as left ventricular hypertrophy and heart failure with preserved ejection fraction.^58^ This may be influenced by poor adherence to antihypertensive medication, which may be particularly prevalent in young women.^59^ In addition, a role for sex chromosomes, sex hormones, and reproductive events may influence the sex differences in blood pressure over a life course.^60^ These data highlight the importance of primary prevention and early detection and intervention of raised blood pressure in young women, and the importance of treatment adherence once hypertension is diagnosed.

**Table 1:** Ischaemic heart disease risk factors particular to young women

	Risk factor	Impact
Traditional factors	Blood pressure (BP)	Leading CVD risk factor in women globally Elevation in BP starts at younger age in women BP raises more rapidly in women than in men Women more often suffer long-term consequences of elevated BP (LVH, HFpEF) Female reproductive events may influence sex differences in BP over a lifetime
	Lipids	Recent data support causal effect of LDL-cholesterol on CVD risk in women Familial hypercholesterolaemia diagnosed later in women, leading to increased health impacts compared with men Limited data on effects of female sex hormones on lipids
	Diabetes mellitus (DM)	DM incidence is rising exponentially globally, including in young adults. Risk of IHD in younger women with DM is higher than in younger men
	Overweight and obesity	Affects over a third of women aged over 20 years globally, predicted to risk to 50% by 2050 Strong inverse relationship between BMI and age at diagnosis of DM Obesity linked to lower educational level and socio-economic disadvantage in women Association between obesity and coronary events in severely obese young women
	Cigarette smoking	Prevalence remains high in younger adults worldwide Incidence in women is static or increasing compared with men Smoking at younger age more than doubles risk of atherosclerotic CVD in women and men E-cigarette smoking is particularly prevalent in younger adults and has detrimental acute CV effects, but long-term effects are yet unclear
Gynaecological factors	Menstrual cycle irregularities	Irregular or long cycles can increase CVD risk
	Ovarian/hormonal dysregulation	
	Premature ovarian insufficiency (POI)	Increases the risk of CVD, related to adverse effects on lipid profile, systolic BP, and insulin sensitivity
	Polycystic ovary syndrome (PCOS)	Associated with insulin resistance and hyperinsulinaemia, coronary calcium and increased carotid intima-media thickness Despite detrimental changes in CVD risk factors, increases in CV events are unclear in the long term Recommended: CV risk assessment, lifestyle modifications, screening for gestational diabetes during pregnancy
	Pregnancy-related disorders	
	Hypertensive pregnancy disorders	Comprised of hypertension preceding pregnancy, gestational hypertension, and pre-eclampsia Pre-eclampsia increases the risk of hypertension and IHD Recommended: self-monitoring of BP early postpartum and CVD risk assessment performed annually
	Gestational diabetes mellitus	Increases risk of DM postpartum and risk of future CV event Recommended: glucose testing soon after delivery then every 1–3 years thereafter
	Preterm delivery	Associated with increased CVD risk factors, CAD events, and death Recommended: counsel patients to optimize risk factor profile
	Spontaneous pregnancy loss	Increased risk of IHD independent of subsequent development of CVD risk factors

IHD, ischaemic heart disease; CVD, cardiovascular disease; BP, blood pressure; LVH, left ventricular hypertrophy; HFpEF, heart failure with preserved ejection fraction; LDL, low-density lipoprotein.

The rate of hypercholesterolaemia has increased globally over the past 30 years; however, the age-standardized rate has decreased, with almost 40% of disability-adjusted life years caused by hypercholesterolaemia being attributed to women.^61^ LDL-cholesterol was thought less important as a CVD risk factor in women however recent evidence supports a similar causal effect of LDL-cholesterol on CVD risk in women and men.^1^ In the younger population, familial hypercholesterolaemia is often responsible for elevated LDL-cholesterol concentrations.^62^ Women with familial hypercholesterolaemia are diagnosed later and are undertreated, and the relative impact of the condition on CV risk is higher in women than in men.^63,64^ Overall, there is limited information on lipids, women and female sex hormones, leaving an obvious area for further future study.

Globally, the incidence of diabetes mellitus (DM) has quadrupled in the past 30 years, occurring in about 1 in 11 adults, with obesity, sedentary lifestyle, and unhealthy diet considered important drivers of this increase.^65^ Likewise, diabetes in young adults has increased exponentially; the International Diabetes Federation reported an estimated 63 million young people (aged 20–39 years) in 2013, elevating to 260 million in 2021.^66,67^ Diabetes nearly doubles the excess risk of vascular diseases independent of other risk factors, and the risk of IHD is higher in younger women (aged 40–59 years) with diabetes than in their male counterparts.^68^

Overweight and obesity affect over a third of women (1.11 billion in 2021) and a third of men (1 billion) aged over age 20 years worldwide, a large proportion living in China, India, and the USA, with estimates forecasting that over half the global adult population will be overweight or obese by 2050.^69^ Lower educational level and social disadvantage are associated factors particularly affecting women.^70,71^ Ischaemic heart disease risk factors type-2 diabetes, hypercholesterolaemia, and hypertension are associated with obesity,^72^ and the prevalence of obesity is greater in younger diabetics than older adults with type-2 diabetes.^66^ Long-term data in young women are scarce; however, one prospective study reported a marked increased risk of early CVD outcomes in severely obese young women compared with lean young women, including a four-fold increased risk of acute myocardial infarction at 20-year follow-up.^73^ As a leading avoidable IHD risk factor, urgent action is needed to reduce obesity’s effects on cardiovascular morbidity and mortality.

Cigarette smoking is a well-established risk factor for CVD.^74^ Whilst smoking prevalence has reduced in Western countries, smoking trends remain elevated in developing countries and in younger adults, with e-cigarettes fuelling interest in new smokers particularly in the younger generation.^75,76^ Although the detrimental acute effects of e-cigarettes and their constituents on the cardiovascular system are known, the long-term effects of e-cigarette use on CVD risk are not clear.^77^ Initiation of e-cigarettes leads to subsequent cigarette smoking among young adults, an added avenue towards a new generation of cigarette smokers.^78^ Globally, fewer women smoke than men; however, there have been small changes or increases in women smoking compared with a decreased prevalence in men.^74^ In young healthy adults, current smokers have more than double the risk of atherosclerotic CVD than never smokers, similar among women and men.^79^ Ongoing efforts to prevent smoking must continue, particularly targeting the young, in an effort to reduce premature onset of CVDs.

### Gynaecological factors affecting ischaemic heart disease risk

Gynaecological factors affecting IHD risk generally present in the younger years but can have life-long significance. These factors encompass menstrual cycle irregularities, ovarian and hormonal dysregulation, and pregnancy-related disorders through to the menopausal transition (*Table 1*).^80^ Menstrual cycle dysfunction such as irregular or long cycles are associated with increased CVD risk, suggesting that menstrual cycle characteristics may be a useful marker for predicting CVD in women.^81^ An evaluation of differences in CAD risk factors in pre- vs postmenopausal women assessed the relative contribution of menopausal status compared with age in modulating the relationship between hypertension and CAD.^82^ Premenopausal women (mean age 43 years) were more likely to be current smokers, but fewer had a history of hypertension or dyslipidaemia than postmenopausal women.^82^ Despite this lower risk profile, these premenopausal women had a considerable prevalence of CAD—20% had significant epicardial CAD and age-adjusted prevalence of multivessel CAD was comparable in pre- and postmenopausal women who had significant epicardial coronary atherosclerosis.^82^ Systolic blood pressure was found to be a more potent risk factor for CAD in premenopausal women than postmenopausal women, which differs from men where diastolic blood pressure is the strongest predictor of CAD risk.^82^

Loss of ovarian function before aged 40 years, termed primary ovarian insufficiency (POI), increases the risk of CVD, with each year of early menopause reportedly associated with a 3% increased CVD risk.^83^ Associations of POI with CVD risk factors such as adverse lipid profile, systolic blood pressure, and insulin sensitivity may at least partly explain this increase in risk.^80,84^ Women with POI are recommended to take menopausal hormone therapy until the average age of the menopause, for its bone sparing effects and to retard uterine atrophy primarily.^85^ Whether this treatment would reduce cardiovascular risk in these young women remains unclear.^86^

Polycystic ovary syndrome (PCOS) is a common disorder in women, affecting up to 20% of women of reproductive age, which is characterized by ovulatory/menstrual dysfunction, hyperandrogenaemia (excess androgen levels and/or hirsutism), and polycystic ovarian morphology.^87^ Metabolic disturbances, principally insulin resistance and hyperinsulinaemia, are common features resulting in type-2 DM in many women.^87^ In addition, women with PCOS have signs of subclinical CVD such as coronary calcium and increased carotid intimal-medial thickness compared with healthy controls.^88^ Despite this clustering of CVD risk factors however, long-term observational studies of women with PCOS have failed to detect a clear increase in cardiovascular events, possibly due to methodological inconsistencies between studies or/and a normalization of CVD risk factors as PCOS women of reproductive age progress through to the menopause.^87,89,90^ Cardiovascular risk assessment, dietary and lifestyle modifications, and screening for gestational diabetes during pregnancy are recommendations for women with PCOS.^80^

### Pregnancy-related disorders affecting ischaemic heart disease risk

Pregnancy-related disorders that increase IHD risk and future events encompass hypertensive pregnancy disorders, gestational diabetes, preterm delivery, and recurrent pregnancy loss.^91^  *Table 2* summarizes each condition, their impact on IHD risk, and recommendations for management. Awareness of IHD risk among young women who experience these conditions is vital, with education ideally starting in the peripartum period to best prevent future morbidity.^93^ Hypertensive pregnancy disorders include chronic hypertension that precedes pregnancy, gestational hypertension (develops after 20 weeks of gestation and usually resolves within 6 weeks postpartum), and pre-eclampsia which is characterized by sudden-onset hypertension after 20 weeks of gestation together with one other associated complication, such as proteinuria, maternal organ dysfunction, or uteroplacental dysfunction, and is a leading cause of maternal morbidity and mortality worldwide.^94,95^ Guidelines recommend treating all pregnant women with blood pressure measurements of ≥140/90 mmHg to reduce progression to severe hypertension and state that the only cure of pre-eclampsia is delivery, ideally at 37 weeks.^94^ Prior to that, an intensive blood pressure lowering approach using medical therapy is recommended.^94^ Following delivery, women with a history of pre-eclampsia are at increased risk for future hypertension, IHD, and CVD death.^96^ As a result, self-monitoring of blood pressure in the early postpartum period may be of value^97^ and annual CVD risk assessment is recommended.^94^

**Table 2:** Treatment of angina in young women

Characteristic	Treatment
Risk factor management	Particular focus on Smoking cessation Weight management Blood pressure (monitoring/hypertension control)
Anginal symptoms^11^	Tailor to Blood pressure and heart rate Comorbidities Concurrent medications Patient preference Initial therapy Beta-blocker Calcium channel blocker Therapies to add Long-acting nitrates Nicorandil Ranolazine Ivabradine Trimetazidine
Menstrual angina	Hormone-based therapies Low-dose oestrogen/progestin (preferably transdermal), alone or in combination with conventional antianginal therapy
ANOCA/INOCA^11,92^	MVA Combinations of Beta-blockers Calcium channel blockers Ranolazine Nicorandil Ivabradine Trimetazidine VSA Calcium channel blockers Long-acting nitrates Nicorandil Either subtype Angiotensin-converting enzyme inhibitor Angiotensin receptor blocker Statin Alternatives Pain relief Psychological therapies (CBT, hypnotherapy) Cardiac rehabilitation Support groups

ANOCA, angina with no obstructive coronary arteries; INOCA, ischaemia with no obstructive coronary arteries; MVA, microvascular angina; VSA, vasospastic angina; CBT, cognitive behavioural therapy.

Gestational diabetes affects about 7% of pregnancies, and women with a history of gestational diabetes have a significant elevation in risk of DM following delivery.^98^ Future cardiovascular event risk is doubled in women who have had gestational diabetes, becoming evident within the first 10 years following pregnancy.^99^ Women with gestational diabetes display a cluster of CVD risk factors such as hypertension and dyslipidaemia, with observational studies showing increased incident IHD independent of diabetes.^99^ Guidelines recommend glucose testing soon after delivery, with repeat testing every 1–3 years.^80^

Preterm delivery is defined as delivery before 37 weeks of gestation and occurs in 10% of births globally.^100^ Preterm delivery is associated with increased incidence of maternal CVD risk factors and CAD events and death.^101–103^ No specific monitoring is advised in these women beyond counselling to optimize any modifiable cardiovascular risk factors.^80^

Spontaneous pregnancy loss occurring before 20 weeks of gestation affects up to 24% of pregnancies^104^ and is associated with increased IHD risk.^105^ A 24-year follow-up study indicated that women who have experienced pregnancy loss have an increased risk of CVDs such as IHD and stroke, independent of subsequent development of cardio-metabolic risk factors.^106^ Endothelial dysfunction may link recurrent pregnancy loss and CVD although a true causative effect is yet to be proven.^107^

A sign of the importance of gynaecological factors in the assessment of CVD risk is the publication of a consensus document by the European Society of Cardiology (ESC), written by a multidisciplinary group of cardiology, gynaecology, and endocrinology experts, in an effort to guide the clinical community on diagnostic approach and management of cardiovascular health during menopause, pregnancy, and gynaecological conditions.^80^ In a similar vein, the American Heart Association published a Presidential advisory statement advising collaboration of cardiovascular physicians with obstetricians and gynaecologists to optimize early identification and modification of CVD risk factors in women.^108^ The aspiration is to empower women to take responsibility for managing their CVD risk early in their lives, reinforcing preventive measures regularly whenever women attend healthcare visits, with the ambition to reduce risk of future CVD events.

## Treatment of stable angina

Treatment of stable angina should begin with education on risk factors and symptom control as well as counselling on lifestyle interventions. As discussed earlier, risk factor management in young women should focus on smoking cessation and weight management, together with blood pressure home monitoring and/or management of hypertension in those affected or at increased risk. Reducing sedentary time and increasing aerobic activity (150–300 min of moderate intensity) is recommended by the ESC in all patients with established IHD.^11^

Without trials of antianginal/anti-ischaemic medication specifically in young women available, there is no evidence to guide medical management, nor is there strong evidence for conventional antianginal medication in women generally.^109^ In these circumstances, we turn to the guidelines which advise that anginal symptom control should be tailored to the individual’s blood pressure and heart rate, comorbidities, concurrent medications, and preferences, as well as local drug availability.^11^ Initial therapy is often a beta-blocker or calcium channel blocker, with other antianginal drugs added as required.^11^ Anecdotally, menstrual angina or menstrual coronary vasospastic angina has been effectively treated using hormone-based therapies, either alone or in combination with conventional antianginal medications including nitrates,^17,18,26,27^ but we could find no trial data to support this therapy. Clearly, this is an area for future investigation.

Symptom management of ANOCA/INOCA is challenging as the pathophysiology can be heterogeneous, and there is a scarcity of good quality trials. Current management algorithms revolve around subtypes of ANOCA/INOCA, using combinations of beta-blockers, calcium channel blockers, ranolazine, nicorandil, ivabradine, and trimetazidine for management of MVA and calcium channel blockers, long-acting nitrates, and nicorandil to treat vasospastic angina, with statins and angiotensin-converting enzyme inhibitors/angiotensin receptor blockers as alternatives for either subtype.^32,110^ The anti-ischaemic calcium channel blocker diltiazem has been shown to improve symptoms and CMD in patients with ANOCA/INOCA.^92,111^ Alternative treatments such as pain relief, psychological interventions (cognitive behavioural therapy, hypnotherapy), cardiac rehabilitation, and support groups can help alleviate symptoms; however, there is no evidence to support their use specifically in younger women.^56,112–114^ A recent trial has not demonstrated a benefit of the endothelin-A receptor antagonist zibotentan on MVA^115^; however, trial registration databases show that new studies are currently being planned.

Whatever the initial treatment, response should be assessed early and the treatment adapted if symptoms persist or medication is not tolerated. Evidence from young adults with hypertension indicates that adherence to a medical regimen may be challenging in younger individuals, with poor adherence resulting in increased risk for future cardiovascular events.^59^ In addition to medical management, healthcare professionals treating young women with stable angina may seek multidisciplinary advice from specialties such as gynaecology, endocrinology, or pain management specialists if conventional antianginal treatment fails to alleviate symptoms.

## Knowledge gaps and future research

Epidemiology shows that the prevalence of IHD in younger women is increasing; however, there is a scarcity of evidence describing the prevalence and incidence of *angina* in young women. As angina is the primary presenting symptom of IHD and is associated with increased risk of major cardiovascular events in individuals of all ages, it is essential to discover whether these data specifically hold in a contemporary, young female population, to better understand the magnitude and nature of the problem.

The characteristics of presenting anginal symptoms in young women are not well described, so future studies could investigate whether symptom features align with characteristics typical of older women. The body of evidence describing menstrual angina is largely limited to case reports currently, but it provides an intriguing foundation for further clinical trials into the role of the ovarian hormone fluctuations on anginal symptoms in affected young women. Likewise, there is a need for contemporary and confirmatory data to support cyclical changes in myocardial ischaemia in affected young women with stable angina. The effect of ovarian hormones on the coronary and peripheral circulations of premenopausal women is not well studied, nor is their role in the pathophysiology and possibly treatment of stable angina in young women.

Treatment of angina in women is based on trials that enrolled mostly men or did not report their findings by biological sex. This leaves a large evidence gap which can only be filled by definitive research—this may involve research methods that go beyond traditional, but very expensive, randomized clinical trials. Although treatment algorithms exist for patients with ANOCA/INOCA, these are not always based on strong evidence. Further research is needed to discover the best treatment options for ANOCA/INOCA patients which may entail conducting larger and longer prospective studies.

More data are needed to better understand the long-term health effects of IHD risk factors specifically in young women. Discovery of innovative ways to enlighten young women of their risk of IHD and encourage them to assume responsibility for their future cardiovascular health should benefit the individuals concerned but also potentially reduce future demand on healthcare systems and costs in the long term.

## Conclusions

Ischaemic heart disease prevalence is increasing in younger women across the globe. Angina is the primary presenting symptom of IHD, and evidence indicates an increased future risk of dying from IHD in young women with angina. Presentation of stable angina in young women appears similar to that in older women; however, symptoms and myocardial ischaemia may occur cyclically in line with the menstrual cycle. Confirmation of whether ovarian hormones are involved in the pathophysiology of stable angina in young women, or could be effective in its treatment, is feasible and is an intriguing area for future study. As well as significant traditional IHD risk factors of importance to young women, awareness of the importance and relevance of the negative association between gynaecological dysregulation and pregnancy-related disorders on future IHD risk must be increased; these are conditions that occur relatively early in a woman’s life that can have detrimental life-long cardiovascular consequences. The aspiration is to quell the concerning upward trends in stable angina and IHD in young women by increasing awareness of risks factors, performing quality research to fill gaps in the evidence and thereby improve the future cardiovascular health for young women.

## Declarations

### Data Availability

No data were generated or analysed for or in support of this paper.
